# Treatment of multiple sclerosis with rituximab: A Spanish multicenter experience

**DOI:** 10.3389/fneur.2023.1060696

**Published:** 2023-03-07

**Authors:** Francisco Gascón-Giménez, Carmen Alcalá, Lluís Ramió-Torrentà, Paloma Montero, Jorge Matías-Guiu, Irene Gómez-Estevez, Celia Oreja-Guevara, Sara Gil-Perotín, Yolanda Blanco, María Carcelén, Carlos Quintanilla-Bordás, Lucienne Costa, Luisa Maria Villar, Jose Enrique Martínez-Rodriguez, José Andrés Domínguez, Carmen Calles, Inés González, Javier Sotoca, Agustin Oterino, Celia Lucas-Jimenez, Francisco Pérez-Miralles, Bonaventura Casanova

**Affiliations:** ^1^Neuroimmunology Unit, Neurology Department, Hospital Clinico Universitario, Valencia, Spain; ^2^Neuroimmunology Unit, La Fe University and Polytechnic Hospital, Valencia, Spain; ^3^Neurology Department, Dr. Josep Trueta University Hospital, Girona, Spain; ^4^Department of Neurology, Hospital Clinico San Carlos, IdISSC, Madrid, Spain; ^5^Neurology Department, Clinical Hospital of Barcelona, Barcelona, Spain; ^6^Neurology Department, General University Hospital of Valencia, Valencia, Spain; ^7^Neurology Department, Ramón y Cajal University Hospital, Madrid, Spain; ^8^Neurology Department, Del Mar Hospital, Barcelona, Spain; ^9^Neurology Department, Son Espases University Hospital, Palma de Mallorca, Spain; ^10^Neurology Department, Álvaro Cunqueiro Hospital, Vigo, Spain; ^11^Neurology Department, Mutua de Terrasssa University Hospital, Barcelona, Spain; ^12^Neurology Department, Marqués de Valdecilla University Hospital, Santander, Spain; ^13^Systems and Applications Engineer Department, Subdirectorate of Information Systems Hospital La Fe, Valencia, Spain

**Keywords:** multiple sclerosis, relapsing-remitting multiple sclerosis, progressive multiple sclerosis, treatment, rituximab, anti-CD20 monoclonal antibody

## Abstract

**Introduction:**

Rituximab (RTX) is considered a potential therapeutic option for relapsing-remitting (RRMS) and progressive forms (PMS) of multiple sclerosis (MS). The main objective of this work was to investigate the effectiveness and safety of rituximab in MS.

**Patients and methods:**

Observational multicenter study of clinical and radiological effectiveness and safety of rituximab in RRMS and PMS.

**Results:**

A total of 479 rituximab-treated patients were included in 12 Spanish centers, 188 RRMS (39.3%) and 291 (60.7%) PMS. Despite standard treatment, the annualized relapse rate (ARR) the year before RTX was 0.63 (*SD*: 0.8) and 156 patients (41%) had at least one gadolinium-enhanced lesion (GEL) on baseline MRI. Mean EDSS had increased from 4.3 (*SD:* 1.9) to 4.8 (*SD*: 1.7) and almost half of the patients (41%) had worsened at least one point. After a median follow-up of 14.2 months (*IQR:* 6.5–27.2), ARR decreased by 85.7% (*p* < 0.001) and GEL by 82.9%, from 0.41 to 0.07 (*p* < 0.001). A significant decrease in EDSS to 4.7 (*p* = 0.046) was observed after 1 year of treatment and this variable remained stable during the second year of therapy. There was no evidence of disease activity in 68% of patients. Infusion-related symptoms were the most frequent side effect (19.6%) and most were mild. Relevant infections were reported only in 18 patients (including one case of probable progressive multifocal leukoencephalopathy).

**Conclusion:**

Rituximab could be an effective and safe treatment in RRMS, including aggressive forms of the disease. Some selected PMS patients could also benefit from this treatment.

## 1. Introduction

Multiple sclerosis (MS) is a chronic disease that profoundly alters both cellular and humoral immune systems (1). Several lines of evidence suggest that anti-central nervous system (CNS) antibodies or myelin-related glycoproteins might play a major role in the pathogenesis of the disease, as demonstrated by the intrathecal synthesis of immunoglobulins restricted to the CNS (2–4). B cells are also present in MS lesions and meninges and contribute to disease progression through antibody-dependent and independent mechanisms (5).

Along this line, increasing attention is being paid to anti-CD20 monoclonal antibodies (MoAb) capable of destroying B cells for the treatment of MS, conventionally treated with cellular immunity strategies (6–9).

Rituximab (RTX) was the first anti-CD20 MoAb tested in MS by several groups in both relapsing-remitting MS (RRMS) and progressive MS (PMS). RTX has shown high efficacy in relapses and gadolinium-enhanced lesions (GEL) on MRI in both groups and also less progression of disability in a specific subgroup of primary progressive MS (PPMS) patients, younger than 51 years old with at least one GEL (10–17).

Based on these benefits, RTX has been widely administered off-label to RRMS patients who experience disease activity on the standard therapies, and also in PMS (7, 18–21).

The aim of our study was to describe the effectiveness and safety concerns of RTX treatment in MS, both in patients with RRMS and with PMS.

## 2. Materials and methods

### 2.1. Sample population

Patients with MS selected for this study presented either RRMS or PMS, fulfilled the McDonald diagnosis criteria, and received treatment with RTX between January 2008 and December 2019. Follow-up ended in December 2020.

In all cases, approval was obtained from both the Spanish Medicines Agency of the Spanish Ministry of Health (Agencia Española de Medicamentos y Productos Sanitarios–AEMPS) and the local Ethics Committee of each hospital, as per the Spanish Royal Decree for compassionate use of medicines (RD 1015/2009, June 19th). All patients signed a specific informed consent explaining the potential risks and benefits and, if applicable, potential therapeutic alternatives.

### 2.2. Study design and procedures

We designed a multicenter, retrospective study based on prospectively collected data from seven Spanish MS centers. A common database with predefined criteria for data categorization designed specifically for this study was completed with data from each local database, all of which used the same predefined criteria.

The decision to treat with RTX was agreed among the neurologists of each MS unit, based on the following criteria: (1) in RRMS, patients with suboptimal response to standard disease modifying treatments (DMT) or aggressive disease [≥ 2 relapses in <1 year and score > 2.0 in the Expanded Disability Status Scale–EDSS (22)], in whom currently approved second-line DMTs were not considered a safe option due to a risk of progressive multifocal leukoencephalopathy (PML) or another medical condition; (2) in PMS, if one or more of the following criteria were met: (a) increase of disability since the last year measured by the EDSS; (b) presence of GEL; (c) relapse.

It should be noted that other anti-CD20 MoAbs, such as ocrelizumab, were not available in the period during which the treatment was offered. With regard to progressive forms, ocrelizumab is only indicated in PMS patients under 55 years of age who present GEL on MRI scan (23), so patients with PMS who might benefit from an anti-CD20 treatment at the discretion of the MS neurologist expert and who did not meet these criteria were treated with RTX in compassionate use programs or by special indication.

All patients were evaluated in routine clinical practice. Demographics (age at the first symptom of MS and sex) and retrospective clinical data [presence of IgG or IgM Oligoclonal Bands (OCGB or OCMB)], previous DMT use, EDSS score, and annualized relapse rate [ARR] were collected at baseline (defined as RTX start date).

Both EDSS score and ARR were obtained from the year before RTX and at baseline.

Neurological examination, including EDSS and presence of new symptoms or potential side effects were performed at baseline and every 6 months thereafter, according to clinical practice.

Previous radiological activity was recorded in most patients. An MRI scan including T2-weighted and gadolinium-enhanced T1-weighted sequences were performed at least once a year during RTX administration.

### 2.3. Treatment regimen: Induction and maintenance

RTX induction and maintenance regimens were classified according to the protocols applied at participating centers as follows:

(1) For the induction regimen, all centers administered two 1,000 mg infusions two weeks apart, along with intravenous premedication to prevent allergic reactions to the infusion that consisted of paracetamol, prednisolone, and dexchlorpheniramine. RTX was administered in outpatient facilities by trained nurses. The attending neurologist recorded all infusion-related adverse effects.

(2) Maintenance regimens were classified as follows:

- Re-infusion of a single dose of 1,000 mg based on reappearance of CD19+ (exceeding 1% of peripheral mononuclear cells) or CD27+ memory cells (exceeding 0.05%).
- Fixed re-infusion of a single dose of 1,000 mg every 6 months.
- Fixed re-infusion of a single dose of 1,000 mg every 6 months during the first year and 500 mg every 6 months thereafter.

### 2.4. Definitions

Relapse was defined by the presence of new or worsening neurological symptoms, lasting more than 24 h, in the absence of fever or significant infectious processes and accompanied by objective changes in the neurological examination.

Confirmed improvement in disease (CID) and confirmed worsening of disability (CWD) were defined by a decrease or increase, respectively, of one point in EDSS (if EDSS was <6) or of 0.5 point (if EDSS was 6 or more) persisting after 6 months.

Clinical activity was defined as the presence of relapses and/or CWD and radiological activity was defined as the presence of new T2 and/or GEL on MRI scan.

No evidence of disease activity (NEDA) was defined as absence of clinical and radiological activity, so evidence of disease activity (EDA) was defined by the presence of any activity, whether clinical or radiological.

### 2.5. Outcomes measures

The annualized relapse rate (ARR) and EDSS before and after RTX, and time to first relapse, the time to CDW, and the percentage of patients with NEDA after RTX were the clinical outcome measures.

The radiological outcome measure was MRI activity expressed as presence of new T2 and/or GD+ lesions at brain MRI performed 1 year from baseline according to local clinical practice.

### 2.6. Statistical analysis

SPSS (Statistical Package for the Social Sciences, Chicago, IL, USA) 21.0.v and GraphPad Prism v5.01 were used. Kaplan-Meier survival analysis was used to explore median time to relapse and increased CWD. Univariate and multivariate Cox regression analyses were used to explore potential predictive variables for CWD status after RTX.

Covariates taken under consideration were gender, age, disease duration, EDSS at baseline, and presence of CWD, ARR, and radiological activity from the previous year to RTX initiation.

## 3. Results

### 3.1. Patient baseline demographic and clinical characteristics

Four hundred and seventy-nine patients completed the induction regimen and had at least one subsequent follow-up visit. Of these, 90 patients (18.8%) from Hospital Universitari i Politècnic La Fe (HUPLF) and Hospital Clínic Universitari (HCU) from Valencia (Spain) were previously included in the publication by Alcalá et al. (19). The follow-up of these patients has been updated for this analysis.

Twelve Spanish centers participated in this study, including a total of selected 479 patients (Supplementary Data). Three hundred and eight patients were women (64.3%) and the median age at first symptom of MS was 32 [standard deviation (SD) 11]. Regarding the clinical form of MS, 188 had RRMS (39.3%) and 291 (60.7%) had PMS (211 SPMS and 79 PMS).

The median disease duration from the first symptom of MS to RTX was 11.1 years (range: 7.1–16.3). Patients' baseline demographic and clinical characteristics and separate data according to clinical form are summarized in Table 1.

**Table 1:** Baseline clinical and demographic characteristics of the entire series and of RRMS, SPMS, and PPMS groups separately.

	**Total (*n* = 479)**	**RRMS (*n* = 188)**	**PMS (*n* = 291)**	***P-*value**
Sex F, M (% F)	308,171 (64.3)	48,140 (74.5)	123,168 (57.7)	*p* < 0.001
Age at first symptom	32.0 (11.0)	28.8 (9.5)	34.2 (11)	*p* < 0.001
Clinical form (%) - RRMS - SPMS - PPMS	188 (39.3) 211 (44.1) 79 (16.5)			
Oligoclonal IgG bands (*n* = 333, % positive)	289 (86.8%)	103(88%)	168 (85.4%)	ns
Oligoclonal IgM bands (*n* = 208, % positive)	114 (54.8%)	42 (58.3%)	65 (52.0%)	ns
Previous treatment (SD)	1.8 (1.3)	2.1 (1.3)	1.5 (1.3)	*p* = 0.01
None First-line DMT (IFN/GA) Second-line DMT (NTZ/FGM/ALT) MTZ/Cy Others (AZA, clinical trial)	95 (21.7) 112 (26.2) 143 (33.4) 45 (9.4) 33 (6.9)	18 (11.4) 31 (19.6) 87 (55.1) 11 (7.0)11 (7.0)	77(28)81 (30)56 (20.7)34 (12.6)22 (8.1)	
Age at starting RTX	45.6 (38.8–52.9)	39.3 (32.4–45.3)	49.8 (43.1–56.5)	*p* = 0.04
ARR the year before RTX (*n* = 435)	0.63 (0.8)	1 (0.9)	0.32 (0.6)	*p* < 0.001
% Patients with GEL at baseline (*n* = 379)	41%	49.3%	40%	*p* = 0.02
EDSS the year before RTX (*n* = 408)	4.3 (1.9)	3.2 (1.6)	5 (1.6)	*p* < 0.001
EDSS at baseline (*n* = 433)	4.8 (1.7)	3.7 (1.5)	5.6 (1.6)	*p* < 0.001
% Patients with confirmed worsening of disability before RTX	41.3%	33.5%	46.3%	*p* = 0.08

Comparisons were made between the RRMS and PMS groups, indicating where there are significant differences.

ALT, alemtuzumab; ARR, annualized relapsing rate; AZA, azathioprine; Cy, cyclophosphamide; DMT, disease-modifying treatment; EDSS, expanded disability status score; FGM, fingolimod; GA, glatiramer acetate; GEL, gadolinium enhanced lesion; IFN, interferon beta; MTZ, mitoxantrone; NTZ, natalizumab; PMS, progressive multiple sclerosis; PPMS, primary progressive multiple sclerosis; RRMS, relapsing-remitting multiple sclerosis; RTX, rituximab; SPMS, secondary progressive multiple sclerosis.

In summary, despite standard treatment for MS, most patients included had active disease during the year before starting RTX, in the form of relapses, progression of disability, radiological activity, or a combination of these events.

### 3.2. Rituximab effectiveness

The median follow-up time after RTX was 14.2 months (IQR 6.5–27.2) and the median time between infusions was 9.8 months (range: 5.9–25.1). The frequency distribution of the duration of follow-up was 22.8% patients 6 months, 21.4% between 6 and 12 months, 26% between 12 and 24 months, and 29.8% over 24 months.

**Figure 1:**
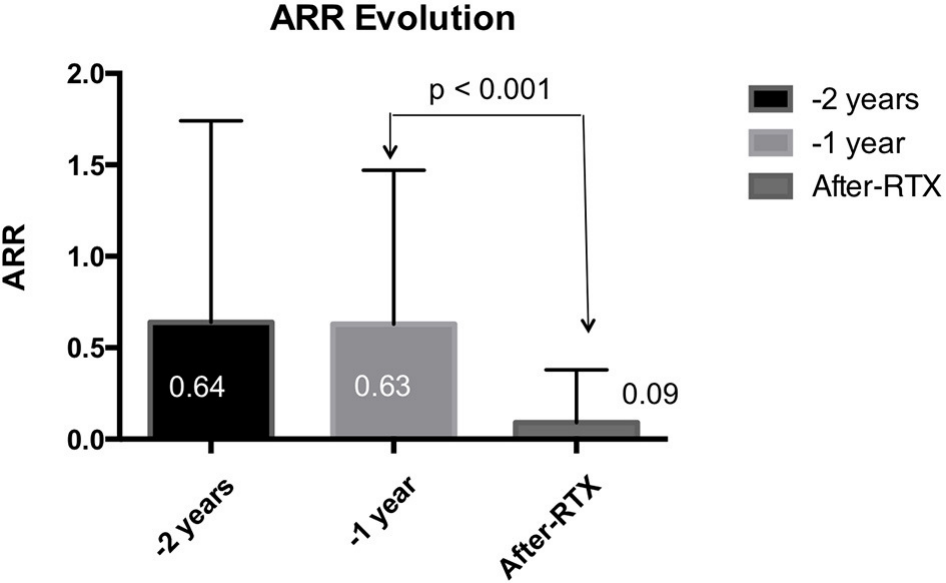
Annualized relapse rate, before (2 years and 1 year) and after starting rituximab. Data for the entire series are shown. A significant reduction in ARR was observed after rituximab infusion. ARR, annualized relapsing rate.

**Figure 2:**
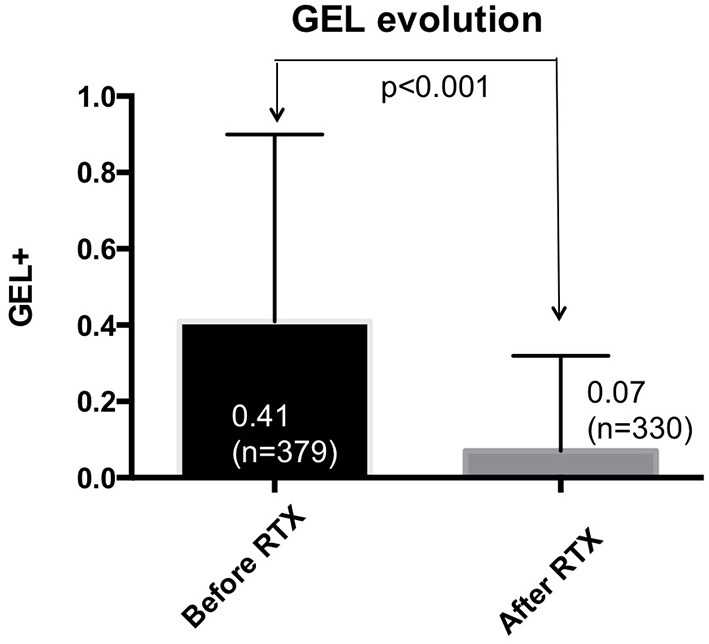
Gadolinium-enhanced lesions, before and after starting rituximab. Data corresponding to the entire series are shown. A significant reduction in GEL was observed after starting rituximab. GEL, gadolinium enhanced lesions.

Taking into account the entire cohort of patients, ARR fell by 85.7% (*p* < 0.001) with respect to the previous year, and there was also a significant reduction in GEL of 82.9% from 0.41 to 0.07 (*p* < 0.001) (Figures 1, 2). Once RTX was initiated, only 45 patients (9.8%) suffered a relapse, half of which (59.1%) occurred within 6 months of starting treatment. Thus, 90.6% of patients presented no new relapses and 93% of patients presented no new GEL after RTX.

**Figure 3:**
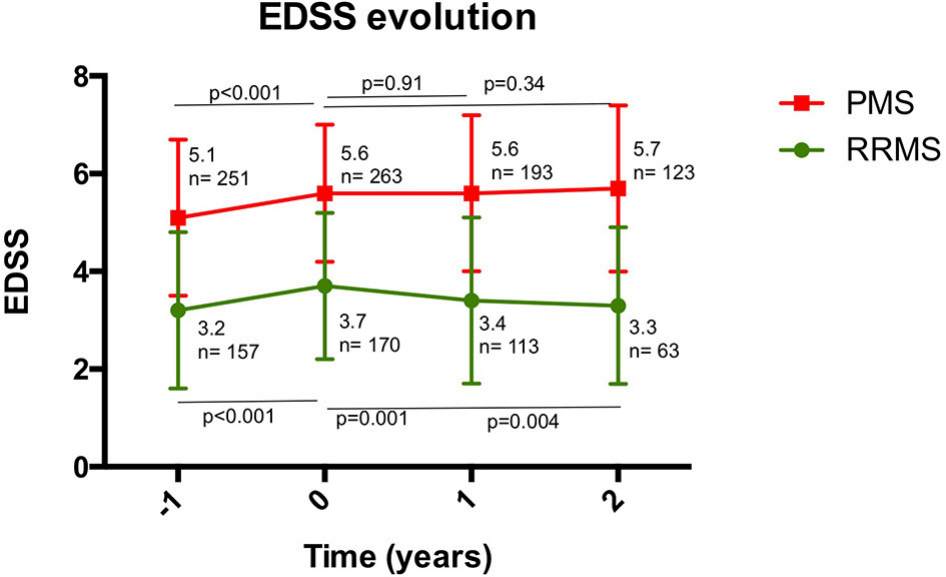
Disability measured by EDSS, before and after the first and second year after starting rituximab. A significant increase in EDSS was observed the year before starting rituximab. For RRMS, a significant decrease in EDSS was observed after the first year of treatment, which was maintained during the second year. For PMS, EDSS remained stable for 2 years after starting rituximab. PMS, progressive multiple sclerosis; RRMS, relapsing-remitting multiple sclerosis.

The mean EDSS score of the overall patient cohort fell from 4.8 to 4.7 (*p* = 0.046) after 1 year of treatment with RTX and remained stable in the second year of therapy. It should be emphasized that the mean reduction in EDSS was more significant in the RRMS subgroup compared to the PMS group, where it remained stable. In the overall cohort, 76.3% of patients did not experience CDW. EDSS variations in each RRMS and PMS are reflected in Figure 3.

There was no evidence of disease activity measured by NEDA in 68% of the total sample, i.e., 74.5% of RRMS patients and 63.8% of PMS patients.

**Figure 4:**
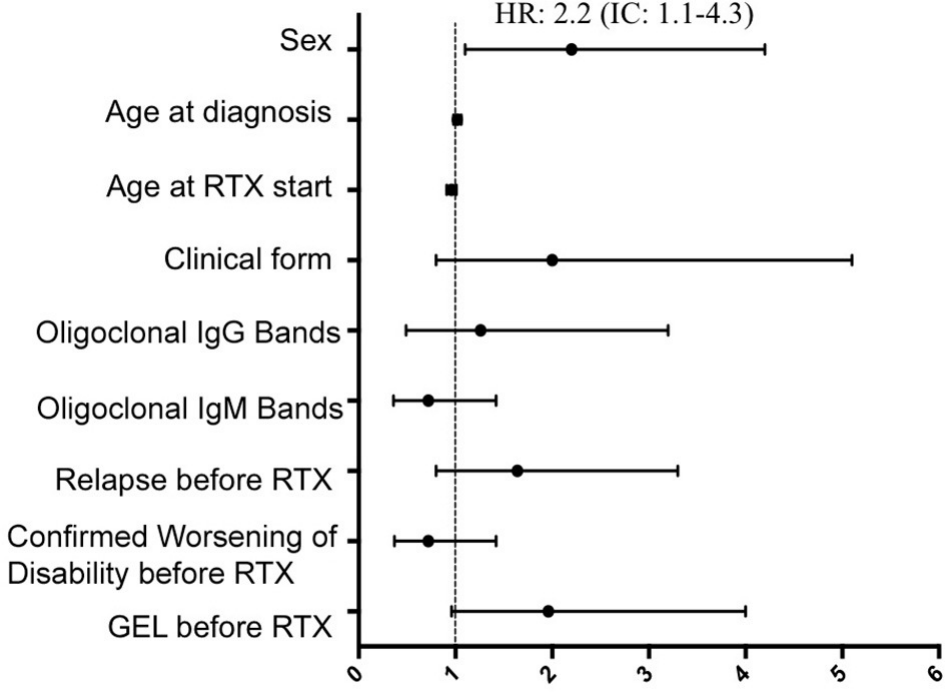
Multivariate Cox regression for predictive variables of time to confirmed worsening of disability in the entire series. Risk for CWD status after RTX was greater in male patients. CWD, confirmed worsening of disability; GEL, gadolinium-enhanced lesions; RTX, rituximab.

**Figure 5:**
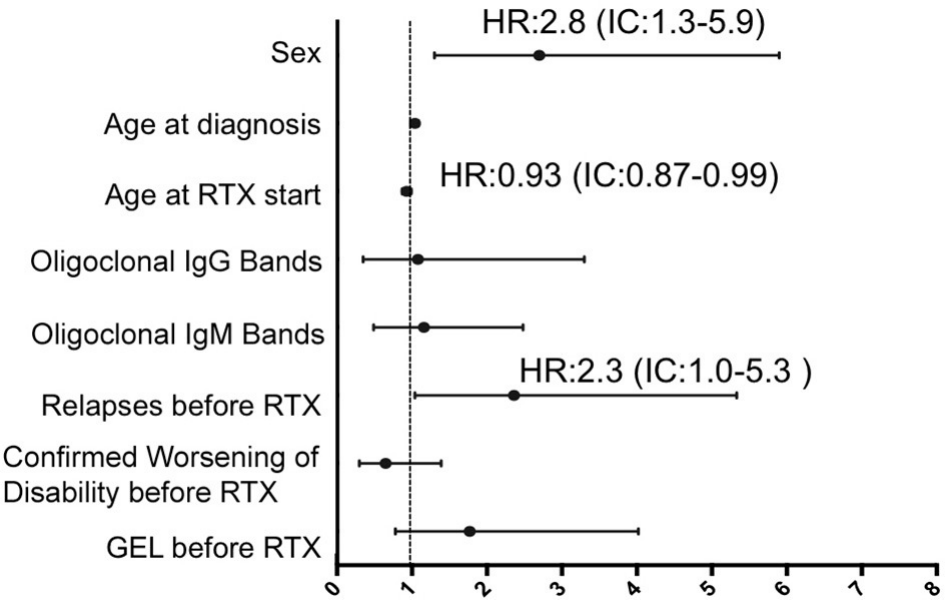
Multivariate Cox regression for predictive variables of time to confirmed worsening of disability in progressive multiple sclerosis patients. Male sex, a younger age at baseline, and absence of previous relapses were the variables associated with reaching CWD status in this subgroup. CWD, confirmed worsening of disability; GEL, gadolinium-enhanced lesions; RTX, rituximab.

In the multivariate Cox regression, risk of CWD in all patients after RTX was higher in male patients (hazard ratio 2.2, CI: 1.1–4.2, *p* = 0.01), and a trend was also observed in patients without GEL in a previous MRI scan (hazard ratio 1.9, CI: 0.9–5.4, *p* = 0.04). Selecting only patients with PMS, a younger age at baseline (hazard ratio 0.9, CI: 0.87–0.99, *p* = 0.05), absence of previous inflammatory activity in form of relapses (hazard ratio 2.4, CI: 1.1–5.3, *p* = 0.04), and again male sex (hazard ratio 2.8, CI: 1.3–5.9, *p* = 0.008) were the variables related to CWD. Data are shown in Figures 4, 5.

When analyzing the patients that used RTX as a first-line treatment vs. escalation from other DMT (22 and 78%, respectively) no differences were found in CWD, radiological activity and NEDA (*p* = 0.44, *p* = 0.65 and *p* = 0.67). However, fewer patients that used RTX as first line treatment experienced relapses (1.1 vs. 11%, *p* = 0.03) with a trend toward a longer time to relapse (80 vs. 86.8 months, *p* = 0.07).

Regarding RTX maintenance regimens, 83.7% received 1,000 mg single dose re-infusions based on CD19 or CD27 reappearance, 11.1% received fixed 1,000 mg single dose re-infusions every 6 months, and 5.2% received fixed re-infusion of a single dose of 1,000 mg every 6 months during the first year and 500 mg every 6 months thereafter. No differences on ARR (0.09, 0.15, 0.04, *p* = 0.23), GEL (0.07, 0.09, 0.04, *p* = 0.77), CWD (22, 23, 42%, *p* = 0.09) or NEDA (58, 44, 43%, *p* = 0.15) were found between groups.

### 3.3. Rituximab tolerability and safety

The main side effect of RTX was the appearance of infusion-related symptoms at first infusion (77 patients, 19.6%), despite premedication with antihistamines, steroids, and acetaminophens. Most of these symptoms were mild (85.9%) and were resolved by decreasing the infusion rate. Only in two patients was RTX definitively discontinued due to moderate infusion-related symptoms, namely generalized rash and symptomatic bradycardia. Three patients suffered symptoms consistent with serum sickness that were managed successfully with symptomatic treatment, but RTX was no longer administered.

During follow-up, 18 patients (3.7%) reported a significant infection with good recovery with standard treatment. Regarding RTX different maintenance regimens no differences on significant infections (2.2, 3.8, and 4%, *p* = 0.29) were found between groups. COVID-19 infection was not registered specifically, as follow-up period was done until December 2020. However, only 17 patients (3.5%) experienced significant urinary or respiratory infections, and no deaths due to COVID infection were registered. One patient developed probable progressive multifocal leukoencephalopathy (PML) while receiving RTX. This was a 57-year-old man with PPMS and EDSS score of 7 who presented new progressive symptoms (motor and cognitive) with EDSS score worsening to 7.5, and lesions on MRI suggestive of PML 26 months after RTX. Although determination of JC virus in CSF was negative, PML diagnosis was considered after excluding other alternative diagnoses based on typical clinical and radiological findings. RTX was stopped and mefloquine and mirtazapine were administered, with periodic intravenous immunoglobulins, resulting in clinical and radiological improvement, with recovery to EDSS 7 without clinical sequels. One patient with PPMS and severe disability (EDSS 6.5) suffered a serious urinary infection with associated sepsis and died.

One patient presented agranulocytosis 3 months after RTX infusion. After infection and other toxic substances were ruled out as responsible for the decrease in neutrophils, this side effect was attributed to RTX and the treatment was permanently discontinued.

Three patients experienced venous thrombotic events. One developed an isolated deep venous thrombosis (DVT) in one leg, another developed a DVT with secondary mild pulmonary embolism; both patients continued RTX treatment. The second patient was taking concomitant oral contraceptives that were withdrawn. The third case had a serious massive pulmonary embolism due to a DVT that led to sudden death. This latter case had an EDSS score of 8.5, so lack of mobility could have contributed to venous stasis. No new appearance of neoplasms was observed. Main side effects are summarized in Table 2.

**Table 2:** Main side effects and withdrawals for this reason.

**Side effects**	N (^*^)	Withdrawal (^**^)
Infusion-related symptoms	77 (19.6%)	2 (2.6%)
Serum sickness	3 (0.62%)	3 (100%)
Significant infection	18 (3.7%)	4 (22.2%)
- Respiratory or urinary infections	17 (3.5%)	3 (17.6%)
-PML	1 (0.2%)	1 (100%)
Toxic agranulocytosis	1 (0.2%)	1 (100%)
Venous thrombotic events	3 (0.6%)	1 (33.3%)
Total	102 (24.3%)	11 (10.8%)

PML progressive multifocal leukoencephalopathy; ^*^Percent of patients of the entire sample; ^**^Percent of patients of all patients with this side effect.

### 3.4. Withdrawal of rituximab treatment

RTX was discontinued in 61 patients (12.7%). CWD was the reason for withdrawal in 21 patients (32.8%), all of whom had PMS. In five patients, RTX was withdrawn due to inflammatory activity (four patients presented relapses and one patient had isolated radiological activity). Three of these patients underwent autologous hematopoietic stem cell transplantation and the other two were switched to ocrelizumab. In 11 cases (18%), RTX was discontinued due to side effects: infusion-related symptoms (2 patients), serum sickness (3 patients), agranulocytosis (1 patient), recurrent infections (2 patients), severe urinary sepsis (1 patient), PML (1 patient) and thrombotic events (1 patient). Four patients stopped RTX when they wished to become pregnant. RTX was switched to ocrelizumab, once it became available, in 7 patients who met the criteria for this drug. RTX was discontinued according to the criteria of their neurologists, who believed that the risk of side effects exceeded the benefits of the treatment (stable patients with high disability). Finally, three patients were lost to follow-up.

## 4. Discussion

In this cohort of MS patients that included individuals with aggressive disease who were followed for a mean period of 14.2 months, RTX was well-tolerated, safe and useful for controlling inflammatory activity and the short-term progression of disability in patients with RRMS and selected patients with PMS. RTX helped achieve NEDA status in both RRMS and PMS patients.

Four clinical trials have evaluated the efficacy and safety of rituximab: three in RRMS and one in PPMS (8, 11, 13, 16). These studies are very heterogeneous in their design, outcomes, and infusion protocols, but all authors conclude that rituximab is effective in reducing clinical and radiological activity in RRMS. The OLYMPUS trial in PMS showed that RTX marginally reduced the time to CWD status, but the difference did not reach statistical significance except in a preplanned subgroup of young patients (<51 years of age with GEL in the baseline MRI) (13). Moreover, recently the multicenter phase 3 RIFUND-MS study has shown that RTX is superior to dimethyl fumarate in preventing relapses over 24 months in patients with early relapsing-remitting multiple sclerosis (24).

Furthermore, multiple observational studies have showned effectiveness of rituximab in reducing disease activity in patients with MS (7, 20, 21, 25–27). Of these studies, the ones with the largest sample size were those by Salzer et al. (7), Zecca et al. (20), Alping et al. (21), Granqvist et al. (26), and Starvaggi et al. (27), with a sample size of 822, 718, 355, 259 and 120 patients, respectively. Interestingly, our study includes 479 MS patients treated with RTX and, to our knowledge, is one of the largest observational studies of RTX in MS.

Similarly to other large real-world studies, results from our series confirm RTX safety and efficacy in RRMS. EDSS reduction was less significant in PMS than in RRMS, but most of our PMS patients remained stable 2 years after RTX and showed no significant changes in their EDSS score. It should be noted that the year before RTX, nearly half of the PMS patients had experienced CDW (41.2%) and after starting RTX, this figure fell to 29.4%, so 70.6% of PMS patients achieved a CWD-free status.

In our series, male sex predicted a poor outcome, increasing the risk of CWD. If we look only at PMS, male sex, younger age at baseline, and the absence of previous inflammatory activity in form of relapses increased the risk of CWD.

In the classical studies of the natural history of MS, both male sex and younger age at onset imply a poor prognosis, and are predictors of progression to irreversible disability in MS, irrespective of DMT use (28). As observed in the OLYMPUS trial, absence of previous inflammatory activity may imply a worse prognosis in PMS patients, increasing the risk of CWD after RTX. This observation might suggest that RTX is more limited in preventing inflammatory-independent degeneration.

In a larger real-world study that included 822 MS patients treated with RTX off-label (557 RRMS and 198 PMS) with a mean follow-up of 21.8 months, similar data to our series were observed (7). A significantly lower ARR was observed in all subgroups, falling to 0.044 for RRMS, to 0.038 for SPMS, and to 0.015 for PPMS, and only 4.6% of patients experienced some radiological activity. In our study, an important decrease in inflammatory activity was also observed for both RRMS and PMS patients, with 90.6% of patients free of relapses and 93% free of radiological activity.

In the above-mentioned study, the EDSS score remained unchanged in patients with RRMS but increased by 0.5 and 1.0 in patients with SPMS and PPMS, respectively. However, the results in our series are more favorable, and no significant increase in EDSS score was observed after RTX for any group, even in PMS patients where it remained stable.

Another large real-world multicenter experience conducted in Italian and Swiss centers in 355 MS patients reported RTX effectiveness and safety data, also showing results consistent with ours (20). In this study, a significant decrease in ARR was observed for RRMS, SPMS, and PPMS. Percentages of patients with a confirmed EDSS progression were 14.6% in the RRMS group, 24.7% in the SPMS group, and 41.5% in the PPMS group. We found similar data in our study, with 7.4% of RRMS, 29.4% of SPMS, and 43.5 % of PPMS patients experiencing CWD.

Regarding the treatment strategy, similar to other cohorts, in our series RTX showed effectiveness both used as first-line treatment or escalation (29). Naïve patients experienced fewer relapses with no difference in CWD. Early use of high efficacy treatment rather than escalation has shown better outcomes in MS (30). However, longer follow-up is needed in our series to assess whether the escalation approach compared with early RTX use may be inadequate to prevent long-term outcomes, including the risk of developing SPMS.

In clinical trials and real-world studies, like ours, the most common side effects were related to the infusion and infections (especially urinary and respiratory), with low cases of serious events. The evidence on the long-term use of RTX in other clinical conditions, such as rheumatoid arthritis, has supported its favorable safety profile (31, 32). However, we still recommend close monitoring to prevent infections, in particular, reactivation of tuberculosis and hepatitis B. Total serum immunoglobulins should also be determined before starting RTX and during follow-up (15).

PML cases have been reported in patients with lymphoma and other inflammatory diseases such as rheumatoid arthritis treated with RTX; however, the JC viral reactivation probably was due to the immunosuppression related to the disease or other concomitant immunosuppressive therapies (32, 33). Recent observational data from over 100,000 MS patients in the FDA Adverse Event Reporting System database indicate that RTX-treated patients have an increased PML risk with an adjusted odds ratio = 3.22 (95% confidence interval: 1.07–9.72) (17, 34). Recently, in the nationwide registry-based cohort study conducted in Sweden, one case of RTX-related PML was described, but the patient had switched from natalizumab within 6 months before PML was diagnosed (35). Here we report one possible case of PML in a patient with MS who had not been previously treated with any other immunosuppressive drug. To our knowledge, this is the first case of PML in a patient receiving RTX for MS without previous other treatments.

Due to the lack of formal dose-finding trials of different RTX therapy regimens, different RTX therapy regimens were used in this study, but no differences in effectiveness or safety concerns were found. A recent study suggests that relapse risk remains low with extended infusion intervals of RTX (27). However, further studies are needed to optimize the dosing regimen and to identify the dosing interval that could possibly be individualized by adjusting to immunological parameters and disease activity. It may be interesting to investigate if a reduced dosing schedule adjusted to CD19 cell concentrations or immunoglobulin replacement can reduce the risk of infections, while preserving efficacy and the favorable safety profile.

Noteworthy, both ocrelizumab (another anti-CD20 MoAb currently approved for MS treatment in both relapsing MS and active PPMS) and siponimod (another currently available treatment for active SPMS patients) had not been officially approved at the time when most of the patients selected for this study began RTX treatment (36–38). For progressive forms, ocrelizumab is only indicated in PMS patients under 55 years of age who present GEL on MRI, so patients with PMS who might benefit from an anti-CD20 treatment and did not meet these criteria were treated with RTX in compassionate use programs or by special indication.

The off-label use of RTX with infusions of 1–2 g annually is less expensive than most of the currently available FDA-approved DMTs, so another advantage of RTX, in addition to efficacy and safety, is that it is a cost-effective therapy.

The main limitations of this study are the observational design, the absence of a control group, and the short follow-up time. The absence of a control group does not allow for confirmation of whether the reduction in clinical and radiological activity observed in our study is actually due to the effect of RTX or to the natural course of the disease or the effect of regression to the mean (39). Nevertheless, the magnitude of the observed reduction in inflammatory activity, even higher than observed in clinical trials, deserves consideration.

These limitations mean that prevention of CWD in PMS particularly needs to be explored in greater depth in large prospective and controlled studies, taking into account specific clinical variables, such as age, disease duration, comorbidities, evidence of inflammatory activity defined by clinical relapses, previous progression rate, and MRI data.

Although we did not compare RTX with other DMTs in our series, real world studies have shown better clinical efficacy of RTX compared to injectable DMTs and dimethyl fumarate and fingolimod, and in some cases even to natalizumab (7, 20, 21, 25–27, 40, 41). Moreover, in patients with PMS off-label RTX has shown similar effectiveness to on-label ocrelizumab (42).

In summary, our study adds to the body of evidence that RTX is effective and relatively safe in the treatment of MS, especially in patients with RRMS.

Although the limitations of this report mean that we cannot provide evidence on its effect on the long-term progression of MS disability, RTX appears to offer a short-term anti-inflammatory effect in PMS patients that is comparable to its effect in RRMS.

## Data availability statement

The raw data supporting the conclusions of this article will be made available by the authors, without undue reservation.

## Ethics statement

The studies involving human participants were reviewed and approved by Ethic Committee of La Fe University and Polytechnic Hospital of Valencia, Spain. The patients/participants provided their written informed consent to participate in this study.

## Author contributions

CA analyzed the data and wrote the manuscript and BC reviewed it. All authors recruited patients from each hospital and have filed the specific database, reviewed, and approved the contents of the manuscript.
