# Supplementary material for: Treatment of multiple sclerosis with rituximab: A Spanish multicenter experience
Source: Front Neurol. 2023 Mar 7;14:1060696. doi: 10.3389/fneur.2023.1060696 (PMC10027934; doi:10.3389/fneur.2023.1060696)
Supplement: Supplementary file 1 [file Table_1.DOCX]

| Hospital | *N* |
| --- | --- |
| Universitari i Politècnic La Fe | 158 |
| Clínico San Carlos | 67 |
| Clínic Universitari, Valencia | 60 |
| Universitari de Girona Doctor Josep Trueta | 53 |
| Clinic Barcelona | 31 |
| General Universitari, Valencia | 25 |
| Universitario Ramón y Cajal | 23 |
| Hospital del Mar | 22 |
| Universitari Son Espases | 19 |
| Álvaro Cunqueiro | 12 |
| Universitari Mútua Terrassa | 5 |
| Universitario Marqués de Valdecilla | 4 |
| Total | **479** |

Supplementary table. Patients selected from each collaborating hospital.
